# Complete genome of *Escherichia* phages Carena and JoYop

**DOI:** 10.1128/mra.01233-23

**Published:** 2024-01-31

**Authors:** Audrey A. Addablah, Solange E. Ngazoa-Kakou, Emmanuella A. Adioumani, Simon J. Labrie, Denise M. Tremblay, Damitha Gunathilake, Sylvain Moineau

**Affiliations:** 1 Plateforme de biologie moléculaire, Institut Pasteur, Abidjan, Côte d'Ivoire; 2 Département de biochimie, de microbiologie et de bio-informatique, Faculté des sciences et de génie, Université Laval, Québec City, Québec, Canada; 3 SyntBioLab Inc., Lévis, Québec, Canada; 4 Félix d’Hérelle Reference Center for Bacterial Viruses, Université Laval, Québec City, Québec, Canada; Queens College Department of Biology, New York, USA

**Keywords:** bacteriophages, *Escherichia coli*

## Abstract

*Escherichia* phages Carena and JoYop were isolated from water samples in Abidjan (Cote d’Ivoire). Their genomes comprise 39,283 and 169,193 bp, encoding 44 and 246 predicted genes, respectively. Carena shares 93.4% nucleotide identity with *Escherichia* podophage CarlSpitteler (*Berlinvirus*), and JoYop shows 95.6% identity with *Escherichia* myophage ADUt (*Tequatrovirus*).

## ANNOUNCEMENT

To establish a phage biobank for biocontrol purposes in Côte d’Ivoire, new phages are being isolated and characterized (1–3). *Escherichia* phages Carena and JoYop were previously isolated from the samples collected in Ebrié Lagoon (latitude 5.356322; longitude −4.091498) and in wastewater of Yopougon (latitude 5.327334; longitude −4.028653), respectively (1). Filtered water samples were added to *Escherichia coli* C in LB (Luria-Bertani) medium and incubated at 37°C for 48 h. Plaques were purified (double-agar layer method) and reamplified with their host in LB at 37°C for 8 h. Observations of phosphotungstic acid-stained phage preparations with a transmission electron microscope (4, 5) revealed that Carena is a podophage and JoYop is a myophage.

DNA was purified from high-titer (>10e^9^ pfu/mL) lysate using phenol-chloroform (6). Illumina DNA Prep kit and IDT 10 bp UDI indices were used for library preparation for genome sequencing. Paired-end reads (2 × 151 bp) were generated with NextSeq 2000 (SeqCenter, Pittsburgh, PA, USA). A total of 10,096,099 and 4,212,638 reads were generated for Carena and JoYop, respectively. Demultiplexing, read quality control, and adapter trimming were performed with bcl-convert (v3.9.3). Assembly was separately performed with Spades v3.13.0 (7) and Ray (8). Both assemblers gave the same contig for each phage. Viral genes were identified with Prodigal v2.6.3 (Prokka) and Glimmer v1.5 (Geneious v11.0.5). Functional annotation was performed with Prokka Galaxy v1.14.5 (9) with NCBI domain searches (10) and blastp (11). Comparisons with other phage genomes were made using NCBI megablast to confirm completeness (11). Tools were run with default parameters.

**Fig 1:**
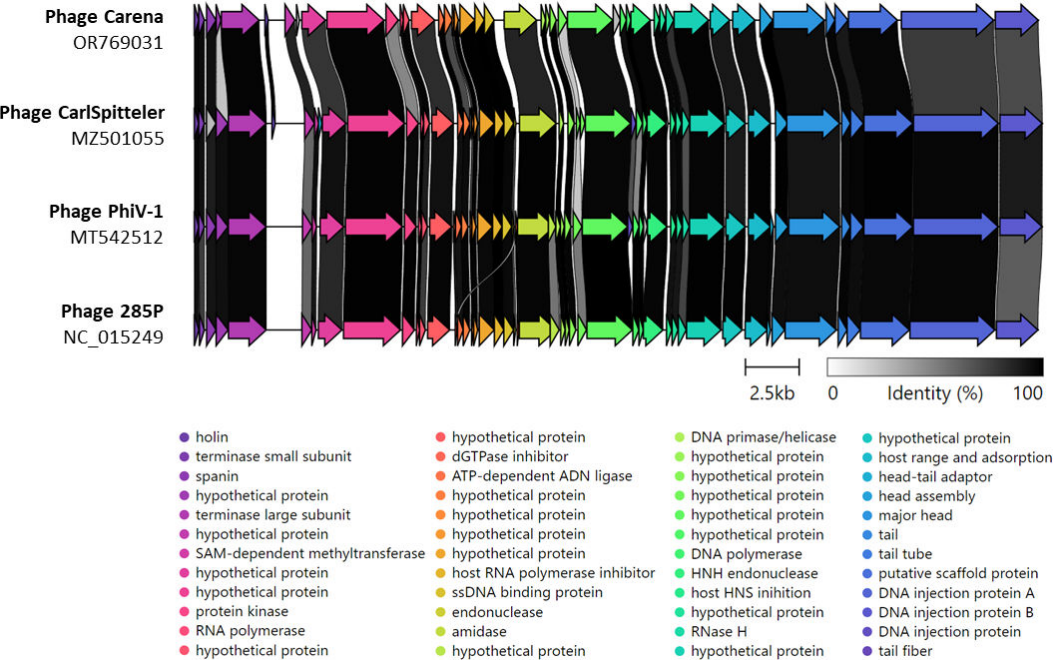
Genome alignment of phage Carena with the genomes of phages CarlSpitter, PhiV-1, and 285P. This analysis was performed using Clinker (15). The identity regions in amino acids are represented in shades of gray.

Carena has a dsDNA genome of 39,283 bp (11,338× coverage), while JoYop contains 169,193 bp (1,741× coverage) with a GC content of 49% and 35.5%, respectively. Homology searches indicated that Carena is related to coliphage CarlSpitteler (Bas68) (12) with an average nucleotide identity (ANI, http://enve-omics.ce.gatech.edu/ani/) of 93.2% and to *Escherichia* phages 285P (ANI 91.2%) (13) and PhiV-1 (ANI 93.2%) (14) (Fig. 1). Thus, Carena belongs to the *Berlinvirus* genus of the *Autographiviridae* family. Forty-four genes were predicted, and 30 deduced proteins (68%) had a predicted function, including proteins involved in DNA replication and packaging, regulation, morphogenesis, and cell lysis. The least conserved proteins are the tail fibers (car1b_044) and two hypothetical proteins (car1b_011 and car1b_026).

Phage JoYop is related to *Escherichia* phages ADUt (ANI 94.7%) (16), vB_EcoM_R5505 (ANI 94.5%) (17), and HP3 (ANI 94.2%) (18). Thus, it belongs to the *Tequatrovirus* genus of the *Straboviridae* family. We predicted 276 genes, including 147 deduced proteins (53%) with predicted functions. Notable proteins include beta-glucosyl-HMC-alpha-glucosyltransferase (cm3_154), alpha-glucosyltransferase (cm3_171), and restriction-modification evasion (cm3_091, cm3_235, cm3_236, cm3_239, and cm3_255) (19). In addition to lysis inhibition proteins rIIA (cm3_110) and rIIB (cm3_109), JoYop encodes for immunity to superinfection (cm3_156) (20). This genome also contains nine tRNAs (Arg, Asn, Tyr, Met, Thr, Ser, Pro, Gly, and Leu), which may compensate for host’s tRNAs due to the differences in GC content or to counteract depletion as an early response to phage infection (21, 22).

## Data Availability

GenBank accession numbers are OR769031 (Carena) and OR769032 (JoYop). SRA numbers are SRR27213143 (Carena) and SRR27213192 (JoYop).
